# Near-Earth object hazardous impact: A Multi-Criteria Decision Making approach

**DOI:** 10.1038/srep37055

**Published:** 2016-11-16

**Authors:** J. M. Sánchez-Lozano, M. Fernández-Martínez

**Affiliations:** 1 University Centre of Defence at the Spanish Air Force Academy, MDE-UPCT. C/Coronel López Peña, s/n. 30720 Santiago de la Ribera, Murcia, Spain

## Abstract

The impact of a near-Earth object (NEO) may release large amounts of energy and cause serious damage. Several NEO hazard studies conducted over the past few years provide forecasts, impact probabilities and assessment ratings, such as the Torino and Palermo scales. These high-risk NEO assessments involve several criteria, including impact energy, mass, and absolute magnitude. The main objective of this paper is to provide the first Multi-Criteria Decision Making (MCDM) approach to classify hazardous NEOs. Our approach applies a combination of two methods from a widely utilized decision making theory. Specifically, the Analytic Hierarchy Process (AHP) methodology is employed to determine the criteria weights, which influence the decision making, and the Technique for Order Performance by Similarity to Ideal Solution (TOPSIS) is used to obtain a ranking of alternatives (potentially hazardous NEOs). In addition, NEO datasets provided by the NASA Near-Earth Object Program are utilized. This approach allows the classification of NEOs by descending order of their TOPSIS ratio, a single quantity that contains all of the relevant information for each object.

Asteroids, described as small rocky bodies with sizes consisting of a few metres to a few hundred kilometres in diameter, constitute a potential threat. While most of them might impact the Earth in the next million years with a probability close to 0.5%^1^, there is a chance of approximately 1% that an impact >1000 MT (equivalent to 100 Tunguskas) might happen once each century^2^. Hence, several studies have explored the implications of large asteroid impacts on early Earth ecosystems^3^,^4^.

It turns out that the determination of the statistical frequency of an asteroid impact is less relevant than stating whether an asteroid may impact the Earth^5^. Even small objects (with diameters ranging from 50 to 100 m) may cause great damage. In fact, these types of small objects may lead to so-called Tunguska-class events whose impact energy is equivalent to 10 MT of TNT^6^.

Near-Earth objects (NEOs) are small asteroids that orbit close to the Earth’s orbit. NEO research provides information concerning the evolution of the early solar system^7^. Several measures to rate hazardous NEOs have been contributed in the scientific literature^8^. Among them are the Torino scale^9^, which evaluates the a priori risk due to a potentially hazardous asteroid, and the Palermo scale^10^, which takes into account the energy at impact and the estimated probability of the event (that might happen in a time period spanning from present time to predicted impact) with respect to a record of events with a comparable or greater level^11^. Several factors, including impact energy, impact velocity, estimated diameter, number of potential impacts, absolute magnitude, and impact probability, are also used to quantify the risk of NEO impacts^10^. Furthermore, other non-physical factors such as Purgatorio Ratio (PR) have been used to manage the communication of impact threats; in this case, the PR is expressed by the ratio of time between the first and last observation to the time between the present and next possible impact date^12^. Therefore, it is clear that an assessment of hazardous NEOs involves a wide list of varied nature criteria. The impact hazard assessment requires the development of ad hoc techniques beyond what is routinely conducted by automatic impact monitoring systems^13^. In this paper, we contribute the first known Multi-Criteria Decision Making (MCDM) approach for hazardous NEO assessment. More specifically, we have applied the Technique for Order Performance by Similarity to Ideal Solution (TOPSIS) to classify hazardous NEOs.

Next, we will motivate the application of MCDM techniques to hazardous NEO assessment. A MCDM problem consists of a set of alternatives to be evaluated with respect to a list of criteria. All that information is contained in a decision matrix. The main goal is to find the best option among all the alternatives once they have been assessed by the decision criteria. Sometimes, it is not possible to find a solution that satisfies all the criteria simultaneously^14^. In these cases, the Multiple Criteria Decision Analysis (MCDA) plays a key role since it allows the decision makers to properly tackle the decision making process. In addition, the MCDA involves a wide range of techniques to systematically address each decision problem. These techniques also facilitate a consensus regarding the final decision and the treatment of a large amount of information, which is usually expressed by various measurement magnitudes and meanings.

Over the years, several MCDM techniques have been developed in the scientific literature. Each MCDM approach uses distinct paradigms and concepts. Among them, we can cite ELimination Et Choix Traduisant la REalité (Elimination and Choice Expressing Reality, ELECTRE)^15^; Analytic Hierarchy Process (AHP)^16^; TOPSIS^17^; Preference Ranking Organization Method for Enrichment Evaluation (PROMETHEE)^18^; Ordered Weighted Averaging (OWA)^19^; Analytic Network Process (ANP)^20^ and VIseKriterijumska Optimizacija I Kompromisno Resenje (VIKOR)^21^. Most of these approaches were introduced during the late nineties, though because of their great usefulness, they are still applied in several areas of engineering such as energy, materials, safety and risk management, operations research and soft computing and information technology management^22^. However, to the best of our knowledge, these techniques have not been previously applied to astronomy and thus constitute the innovation of the present study. In fact, the present study represents the first-known application of MCDM techniques to NEO hazard assessment.

## The decision problem: near-Earth object (NEO) hazardous impact

### Structure of the decision problem

In this paper, we provide a novel scale for assessing hazardous NEOs according to several measures provided by the NASA Near Earth Object Program^23^. We have applied MCDM techniques, including both AHP and TOPSIS approaches, as described in the Methods section. Our main data source consists of the Sentry Risk Table available from the National Aeronautics and Space Administration (NASA) Near Earth Object Program (c.f. Impact Risk Section)^23^. All of the potential future Earth impact events are listed therein once the JPL Sentry System has detected them based on currently available observations. The Sentry System is a highly automated collision monitoring system that continually scans the most current asteroid records and reports the possibility of future Earth impacts over the next 100 years^23^.

Note that all the objects appeared in both tables, namely, both recently observed objects (within past 60 days) and not recently observed objects are sorted in descending order by the Palermo Scale (cumulative). For calculation purposes, we have collected the 101 larger NEOs listed in the Sentry Risk Table on March 16, 2016. These NEOs, which constitute the alternatives of our MCDM approach, were selected as being non-small, i.e., with an estimated diameter >50 *m*. In terms of the Torino Scale, all the objects in the Sentry Risk Table were highlighted in a colour other than white. Thus, following a decreasing Palermo Scale (cumulative) sort order criterion, it follows that the top rated NEOs are 29075 (1950 DA), 101955 Bennu (1999 RQ36), 410777 (2009 FD), 1979 XB, and 99942 Apophis (2004 MN4), as shown on the NASA Near Earth Object Program (Impact Risk) website^23^. The calculations via the MCDM techniques were conducted one day later, on March 17, 2016. This date was taken into account for calculations involving the PR.

Next, we describe the decision criteria involved in the present study. These criteria allow us to calculate a single quantity for each of the 101 potentially hazardous NEOs (alternatives) for classification purposes.

### Decision criteria (C_i_) to assess hazardous NEOs

Our main goal is to assess and classify the potentially most hazardous NEOs. Next, we provide a description regarding all the decision criteria we have selected to undertake this task.

- **C**_**1**_**: Potential Impacts.** The number of dynamically distinct potential impacts detected by the Sentry System. There can be several qualitatively unique pathways that describe an impact in a given year. For example, the pathways can include those having extra revolutions around the Sun or those deflected by an earlier planetary encounter. This criterion must be maximized.
- **C**_**2**_**: Impact Probability (cumulative).** The sum of the impact probabilities from all detected potential impacts. For each potential impact, the corresponding impact probability represents the probability that each tabulated impact will occur. The calculations involving these probabilities are quite complex. This attribute should be maximized.
- **C**_**3**_**: V**_**infinity**_ **(km/s).** The relative velocity at atmospheric entry of the asteroid relative to the Earth, which assumes a massless Earth and disregards the acceleration caused by the Earth’s gravitational field. This quantity is often called the *hyperbolic excess velocity* and is calculated through the expression **V**_**infinity**_^**2**^** = V**_**impact**_^**2**^**−V**_**escape**_^**2**^, where the Earth’s escape velocity is approximately equal to 11.2 km/s. This criterion must be maximized.
- **C**_**4**_**: H (Absolute Magnitude).** This is a measure of the intrinsic brightness of the object. Specifically, it is the apparent magnitude of the object at 1 Astronomical Unit (AU, ~1.4960 · 10^11^ m) from both the Sun and the observer and at a full phase. This attribute should be minimized.
- **C**_**5**_**: Estimated Diameter (km).** The estimated diameter of the asteroid and is calculated under the assumption of a uniform spherical body with a visual albedo equal to 0.154; however, sometimes it is estimated through actual measured values (if available). Nevertheless, since the albedo is rarely known for the objects involved in this study, the diameter estimate is approximate. In most cases, this measure will be accurate to within a factor of two. This criterion must be maximized.
- **C**_**6**_**: Palermo Scale (cumulative).** The cumulative rating of the hazard according to the *Palermo Technical Impact Hazard Scale*^10^. This attribute for the NEOs must be maximized.
- **C**_**7**_**: Energy (Megatons of TNT).** The kinetic energy at impact. This value is based on the absolute magnitude (**C**_**4**_) and impact velocity of each asteroid. The estimation follows the guidelines established by the Palermo Technical Scale and is calculated through the expression **0.5 · Mass · V**_**impact**_^**2**^. The uncertainty in this value is mainly due to the uncertainty in the asteroid mass. Thus, the kinetic energy at impact will generally be accurate to within a factor of three. This criterion should be maximized.

According to the *Summary Table Description* provided for each NEO in NASA Impact Risk Tables, certain parameter values depend on each specific impact event and they seldom change among the different entries. For this reason, only the mean values for the C_3_, C_4_, C_5_ and C_7_ parameters are tabulated.

**Figure 1:**
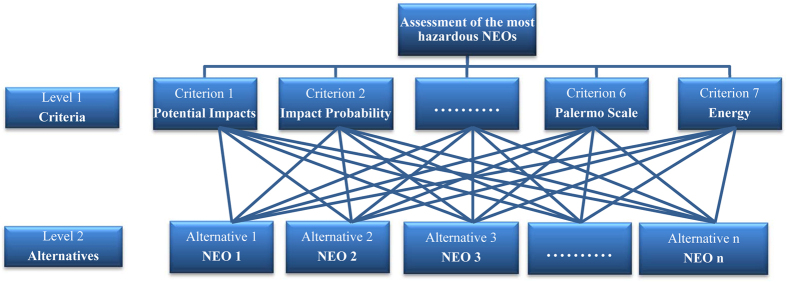
The scheme above represents the hierarchical structure concerning the assessment of hazardous NEOs. The upper node contains the main goal of this paper. Next, the two level MCDM approach is depicted. Specifically, level 1 of the hierarchy contains all the decision criteria, namely, all the NEO features we have considered for classification purposes. The second hierarchical level displays all the alternatives selected to carry out the present study, namely, all the (larger) potentially hazardous NEOs.

A hazardous NEO assessment problem can be understood with a two level, hierarchical structure (c.f. Fig. 1). Consequently, a classifying task for hazardous NEOs becomes a MCDM problem^14^,^17^. Thus, our main goal is to find the best alternative (in this case, the most hazardous NEO) (*A*_*i*_*, i* = *1,…,n* ≥ *2*) regarding all of the decision criteria (*C*_*j*_*, j* = *1,…,m* ≥ *2*) and the experts’ knowledge (*E*_*k*_, *k* = *1,…, r* ≥ *2*). The parameters *n, r*, and *m* are finite numbers.

On the other hand, based on the assumptions of the TOPSIS approach (Methods section), the decision criteria may not be equally important by default. This is the reason a group of four NEO experts from NASA were tasked with determining the relative weights of the evaluation criteria.

### Determining the criteria weights

To determine the weights of the criteria, the group of NASA experts were required to answer a 3-question survey reproduced as follows.

Q1: Do you think that all the decision criteria as described above are equally important to assess and determine which NEOs are most hazardous in the hypothetical case of a potential impact on Earth?

In the affirmative case, it holds that *c*_*i*_ = *c*_*j*_ = *1/n* for all *i and j*. Hence, there is no further action required by experts, since all the criteria weights are the same. However, if there is one expert who believes that all the criteria are not equally relevant, then the following questions must be addressed.

Q2: List the criteria in order of descending importance.

Q3: Compare those decision criteria (Ci) chosen to be in 1st place with those placed in 2nd and so on. The following labels, which correspond to the scale of valuation in the pairwise comparison process, should be used: {(EI), (S + I), (St + I), (VSt + I), (Ex + I)} (c.f. Table 1).

Thus, a pairwise comparison among the criteria was conducted to determine the criteria weights. Then, the criteria weights provided by the experts were unified in the last stage of the alternative assessment. With this aim, a homogeneous aggregation (assuming that the knowledge of each expert is equally relevant across the whole decision process) through arithmetic averaging was completed. Table 2 contains the weights and the rank of each criterion.

Accordingly, the most relevant criterion is C_6_ (cumulative Palermo scale). Interestingly, this criterion has been used by NASA to rank the NEOs in the Sentry Risk Tables^23^ of the Near Earth Object Program. This fact highlights the consistency of the information provided by the experts. The next criteria (sorted by weight in descending importance) were found to be C_1_ (potential impacts) and C_2_ (cumulative impact probability). In contrast, the least important criteria were C_4_ (H magnitude) and C_5_ (estimated diameter).

In addition, using an AHP approach to determine the criteria weights, a consistency analysis of the information provided by experts was conducted. Thus, the consistency ratio was computed by each expert. In all the cases, the consistency ratio measured <0.1 (c.f. Methods section). As a result, we can guarantee the consistency of the applied method to calculate the weights of the criteria, and it was not necessary to revise the judgements provided by the experts.

### Evaluating hazardous NEOs. Results and discussion

Once the criteria set had been selected and their weights quantified, the next step was to provide a measure of the effect caused by each alternative (hazardous NEO) with respect to each criterion. Hence, the TOPSIS approach was used to determine the order of preference among all the alternatives, i.e., the potentially hazardous NEOs with an estimated diameter >50 *m*. Recall that this algorithm is especially appropriate for the assessments of alternatives (on the basis of the criteria) that are not displayed in the same units, as is the case in this study. Each criterion is measured in different units.

Our MCDM approach to assess hazardous NEOs displays all the information in a decision matrix consisting of 101 rows (all the NEOs involved in this study) by 7 columns (all the decision criteria, C_i_). In fact, all the entries of the matrix are quantities provided by the Sentry Impact Risk tables from the NASA Near Earth Object Program^23^.

**Figure 2:**
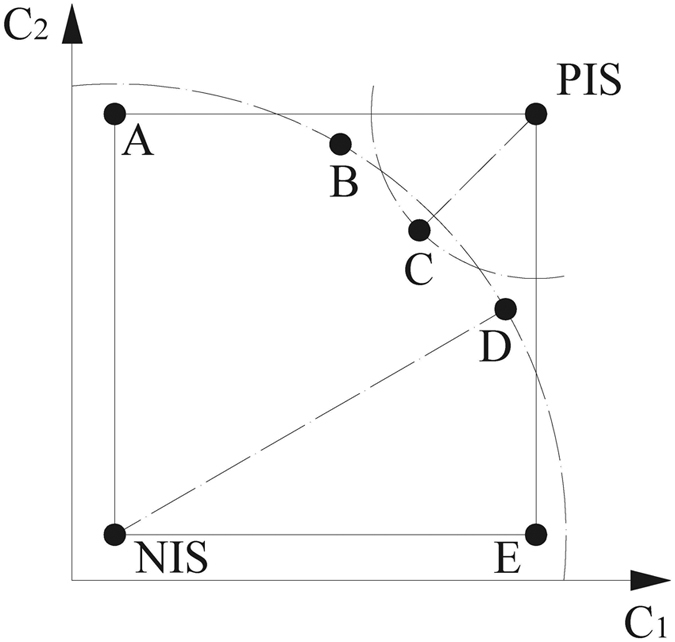
The figure above illustrates the concepts of PIS and NIS in a TOPSIS approach. The optimal alternative is the closest from the PIS (denoted by C) and the farthest from the NIS (located at both B and D points). TOPSIS takes into account the distances to both the PIS and the NIS simultaneously.

Once the TOPSIS approach was applied, a coefficient (*R*_*i*_) for each alternative was obtained according to their relative closeness to the ideal solution (c.f. Fig. 2). Thus, all the NEOs considered in this study have been ranked according to TOPSIS approach. A selection of the 10 most hazardous NEOs ranked in descending order is provided in Table 3. Additionally, Appendix A contains the entire TOPSIS ranking, which includes the 101 NEOs considered in this study.

According to our TOPSIS based ranking, the two most hazardous NEOs are 410777 (2009 FD) and 2011 SR52. These NEOs have the highest TOPSIS scores, which are much higher than the remaining NEOs. The information provided in Table 4 supports the TOPSIS methodology of a continuous balance of either the excess or deficiency in the criteria values. To illustrate this, the 1st ranked NEO 410777 (2009 FD) is considered. The information provided by the Sentry System reveals that this NEO has a low number of potential impacts (criterion C_1_) and low impact energy (C_7_). However, it presents a high value on the (cumulative) Palermo scale (C_6_) and also presents the highest (cumulative) impact probability (C_2_). Likewise, according to the TOPSIS score, the 2nd ranked NEO (2011 SR52) presents only a few potential impacts, has been assigned a low impact probability, and has an intermediate score on the Palermo scale (cumulative). However, 2011 SR52 is the NEO with the highest impact energy. The case of NEO 99942 Apophis (2004 MN4) is quite interesting. This NEO gained a certain notoriety several years ago since it was the first known NEO to reach level 2 in the Torino scale^7^. However, it has been ranked in 19^th^ position by the TOPSIS approach. This is mainly because none of its criteria values are sufficiently relevant. In fact, this NEO presents intermediate values for the most relevant criteria. Furthermore, according to our TOPSIS ranking, the potentially hazardous NEO (101955) 1999 RQ36 in the 7th position has a possibility of colliding with the Earth in the latter half of the 22^nd^ century^24^ and is the primary target of NASA’s Origins, Spectral Interpretation, Resource Identification, Security-Regolith Explorer (OSIRIS-REx) sample return mission^25^,^26^. It has been ranked 7^th^ in the TOPSIS scale for hazardous NEOs since it presents high values for the two most relevant criteria according to the experts, namely, C_6_ (cumulative Palermo scale) and C_1_ (number of potential impacts). Moreover, this object also presents high values for intermediate criteria, including cumulative impact probability (C_2_), absolute magnitude (C_4_) and estimated diameter (C_5_). Finally, we would like to discuss the specific case concerning object (29075) 1950 DA. The impact prediction horizon for this NEO is four times longer than any other asteroid^27^. In fact, the orbit of this object is very precisely known, which allows the possibility to explore centuries in the future, much further than is usually possible (c.f. NASA Near Earth Object website). It is worth noting that (29075) 1950 DA presents the highest values concerning criteria C_6_ (cumulative Palermo scale) and C_5_ (estimated diameter). However, NASA reports only one unique potential impact (criterion C_1_), which moves it to the 10^th^ position in TOPSIS ranking.

## Sensitivity analysis

### Variation of weights

A sensitivity analysis to verify and analyse the validity of the results shown by the TOPSIS algorithm has been conducted. First, we have performed an additional analysis of all the alternatives, i.e., the 101 NEOs considered in our study, but this time under the assumption that all the criteria are equally weighted (equally relevant). Table 5 contains the 10 top-rated hazardous NEOs according to the new TOPSIS ranking.

Although the ranking order does not exactly match the ranking order provided in Table 3, there are only slight differences among the 10 top-rated objects. In fact, under the assumption that all the criteria are equally important, 8 objects still remain among the 10 most hazardous NEOs. Additionally, according to the new TOPSIS ranking, the two first-rated and most hazardous NEOs (410777 (2009 FD) and 2011 SR52) are exactly the same as provided by the previous TOPSIS approach. Only their positions are interchanged. This new approach has allowed the authors to dismiss a possible bias underlying the experts’ knowledge; however, a consistency ratio was previously estimated for this purpose. In addition, the results suggest that the judgements provided by the experts through their AHP based surveys do not greatly influence the criteria weights, and hence the TOPSIS ranking contributed to this paper for hazardous NEOs.

### Involving the Purgatorio Ratio

One of the NASA experts taking part in the AHP survey suggested that the authors utilize the *Purgatorio Ratio* (PR) in the TOPSIS scale for the NEOs. When a NEO is observed during a given time window, this parameter is estimated by the ratio of the first and last observations and the time between the present and the next possible future impact date^12^.

Thus, being encouraged by that interesting proposal, our next goal was to verify the effect of the PR in hazardous NEO assessment. Hence, a new column was added to our decision matrix, which included the PR values for each of the 101 most hazardous NEOs to be assessed. The PR calculations were performed on March 17, 2016 (our present time). Moreover, to determine whether the PR criterion influenced the previous TOPSIS results, we assigned this criterion a value of 25% of the overall relevance. The remaining 75% was equally assigned to the remaining criteria (C_1_–C_7_). Once all the stages involved in the TOPSIS approach were carried out, a new ranking was obtained (c.f. Table 6).

A comparison of the 10 most hazardous NEOs in the TOPSIS ranking with and without the PR criterion (using the weights calculated from the experts’ knowledge) indicates that only three objects are not ranked among the 10 most hazardous NEOs.

In addition, the new first and fourth objects are 2013 NH6 and 99942 Apophis (2004 MN4), respectively. This fact could be justified on the basis that both of them have been observed for a long time (more than 3000 days) and also because the closest impact date is in the far-distant future (2060). Therefore, even if the PR is given a high weight as a decision criterion, we have verified that the actual influence of this parameter in the TOPSIS ranking for hazardous NEO assessment is low.

## Conclusions

In this paper, the first-known MCDM based approach for rating hazardous NEOs has been presented. The steps are summarized as follows. First, we have considered an AHP procedure to quantify the relevance or weight of each criterion involved in our study. For this task, a group of NASA experts was surveyed.

The most relevant criterion was found to be the (cumulative) Palermo scale. This criterion matches the sort order used by the Sentry Risk Tables in the NASA Impact Risk Section (c.f. Near Earth Object Program^23^). A TOPSIS approach was conducted to rate all the involved alternatives, namely, the 101 most hazardous NEOs (with a diameter >50 *m*) according to the NASA Near Earth Object Program. As a result, the two most hazardous objects were found to be 410777 (2009 FD) and 2011 SR52.

Additionally, we have developed a sensitivity analysis to assess whether the experts’ judgements influenced our TOPSIS ranking from a subjective viewpoint and concluded that there was no significant bias. Moreover, our contributed TOPSIS ranking for hazardous NEOs does not substantially vary provided that all the criteria are considered to be equally relevant. This strengthens the results displayed by our MCDM procedures. Similarly, we verified that the PR is not significantly relevant and will not be included as a decision criterion in our study.

Furthermore, the present study has been carried out according to several of the most relevant NEO features identified by the NASA Near Earth Object Program (c.f. Sentry Risk Table and Earth Impact Tables – for each NEO) to assess the hazardous level for these kinds of objects. Additionally, the 101 NEOs involved in our study have been tagged as the most hazardous objects by the NASA Sentry.

Next, we discuss some of the drawbacks regarding the present study. First, our TOPSIS based approach for hazardous NEOs assessment involves several MCDM techniques that should be introduced to astronomers skilled in NEOs. As mentioned above, the MCDM techniques have been widely applied in other scientific fields, though their application to NEO classification is new. Because of this novelty, an understanding of the mathematical basis becomes quite necessary. Another drawback of our TOPSIS based ranking for hazardous NEOs concerns the type of objects considered as the alternatives in our MCDM based approach. More specifically, our TOPSIS ranking for hazardous NEOs is focused on larger objects. This amounts to a total of 101 objects from a larger family^7^.

## Methods

### Analytic Hierarchy Process (AHP)

This methodology has been accepted by the research community as a robust and flexible MCDM model to address complex decision problems^16^. The AHP consists of three main tasks: to structure the complex decision as a hierarchy of goals, criteria and alternatives; to conduct a pairwise comparison of all the elements in each level of the hierarchy with respect to each of the criteria involved in the previous level; and to vertically synthesize the judgements on the different levels of the hierarchy. Therefore, the AHP attempts to estimate the impact of each alternative on the overall hierarchy objective. In this paper, we have applied this methodology for only criteria weight calculation purposes. Next, we summarize how the AHP works.

Let us assume that the quantified judgements provided by the decision maker for any criteria pair (*C*_*i*_*, C*_*j*_) are the entries in the following *n*-order matrix:


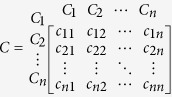


For instance, note that the input *c*_*12*_ is about the relative significance of *C*_*1*_ with respect to *C*_*2*_, i.e., *c*_*12*_ ≈ (*w*_*1*_*/w*_*2*_). That idea can be further extended and hence the statements below can be expressed as follows:

1. *c*_*ij*_ ≈ (*w*_*i*_*/w*_*j*_)*, for all i, j* = *1, 2, …, n.*
2. *c*_*ii*_ = *1, for all i* = *1, 2, …, n.*
3. *If c*_*ij*_ = *α* ≠ *0, then c*_*ji*_ = *1/α, for all i* = *1, 2, …, n.*
4. *If the criterion C*_*i*_ *becomes more relevant than C*_*j*_*, then c*_*ij*_ 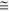 (*w*_*i*_*/w*_*j*_) > *1.*

The abovementioned rules generate a positive and symmetric matrix (*C*) with its main diagonal containing ones. Accordingly, the decision maker provides value judgements to fill in an upper triangular matrix. Moreover, as the Saaty scale indicates^28^, the values assigned to each entry *c*_*ij*_ usually lie in the interval [1,9] or their reciprocals. Therefore, Table 1 contains the decision maker’s linguistic preferences and a pairwise comparison process that have been considered in our hazardous NEO assessment case studies.

Thus, if *n* is the order of matrix *C*, then the number of judgements *L* in the corresponding upper triangular matrix is given by the following expression:


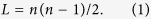


On the other hand, the vector of weights is given by the eigenvector that corresponds to the maximum eigenvalue λ_*max*_ of *C*. The standard eigenvector method to estimate the weights in the AHP methodology *measures* the consistency of the referee’s preferences, which were arranged in the comparison matrix. The Consistency Index (CI) is calculated by *CI* = (λ_*max*_ − *n*)*/*(*n* − *1*), so if the expert shows a minor inconsistency, then λ_*max*_ > *n*. The Saaty scale provides the next indicator for the Consistency Ratio: *CR* = *CI/RI*, where *RI* is the Random Index, which can be calculated as the average value of *CI* (c.f. Table 7) for random matrices^29^,^30^,^31^. Therefore, *CI* is used to quantify the probability that the judgements’ matrix was randomly created^32^ and hence the matrix is said to be consistent (in the sense of Saaty) provided that *CR* < *0.1*.

### TOPSIS approach

The Technique for Order Performance by Similarity to Ideal Solution, known as TOPSIS, was first introduced by Hwang & Yoon^17^ and is one of the classical MCDM procedures^33^,^34^. This approach selects an optimal alternative that presents the shortest distance from the positive ideal solution (PIS) and the farthest distance from the negative ideal solution (NIS). Therefore, the optimal solution provided by TOPSIS constitutes a compromise solution according to the decision-maker’s preferences (c.f. Fig. 2).

The following key arguments support the utilization of TOPSIS in a wide range of applications^35^:

1. TOPSIS logic is rational and understandable.
2. The computation processes are straightforward.
3. The concept pursues the best alternatives for each criterion from an easy mathematical viewpoint.
4. The relevance weights are incorporated into the comparison procedures.

The TOPSIS approach requires several assumptions^36^ to be verified to guarantee the proper application to a given dataset. They are as follows:

1. It is assumed that all the criteria in the decision matrix take either monotonically increasing or monotonically decreasing utility. This means the larger the criterion outcomes, the greater the preference for the “benefit” criterion and a reduced preference for the “cost” criterion.
2. Any outcome expressed non-numerically should be quantified throughout an appropriate scaling technique.
3. By default, all the criteria cannot be assumed equally relevant. In this way, this procedure receives a set of weights from the decision maker.

Next, we provide a detailed description of each step in the TOPSIS approach.

**Step 1:** **Establishing a performance matrix**

*A*_*i*_ (*i* = *1,…,m*) denotes the collection of alternatives that have to be evaluated by the series of criteria *C*_*j*_ (*j* = *1,…,n*). This leads to the following decision matrix (c.f. Table 8):

**Step 2:** **Normalizing the decision matrix**

In this stage, we have obtained the corresponding normalized decision matrix. The value of each criterion is normalized by dividing it by the following (Euclidean) norm.


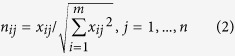


Thus, the scale becomes the same for each criterion.

**Step 3:** **Calculating the weighted normalized decision matrix**

The elements of the weighted normalized decision matrix *V* are calculated through the following expression:


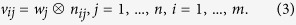


where the *w*_*j*_’s satisfy 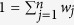. This provides the weight of the jth attribute, which is calculated by the AHP methodology.

**Step 4: Determining the positive ideal solution** (**PIS**)** and the negative ideal solution** (**NIS**)

Let *A*^*+*^ denote the positive ideal value set, which contains the best performance scores, and let *A*^*−*^ be the negative ideal value set, containing the worst performance scores. Mathematically,


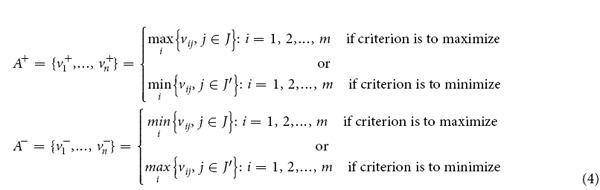


where the index *J* is associated with the criteria that give profits or benefits and the index *J’* is associated with those ones indicating costs or losses.

**Step 5:** **Calculating the separation measures**

To estimate the separation of each alternative (PIS and NIS), the following expressions are calculated:


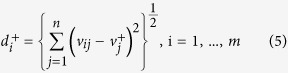



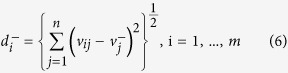


**Step 6: Calculating the relative closeness to the ideal solution**

First, the ranking score *R*_*i*_ is calculated as follows:


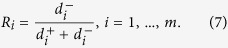


Thus, if *R*_*i*_ = 1, then *A*_*i*_ = *A*^+^, whereas if *R*_*i*_ = 0, then *A*_*i*_ = *A*^−^. Notice that all the *R*_*i*_ lie in the open interval (0,1). Accordingly, if the *R*_*i*_ values are close to 1, then the higher the priority for the *ith* alternative.

**Step 7: Ranking the preference order**

In this stage, we have sorted the best alternatives according to the ranking scores *R*_*i*_in descending order. In this case, the TOPSIS approach was used to evaluate all the alternatives, i.e., the 101 NEOs involved in the present study.

## Additional Information

**How to cite this article**: Sánchez-Lozano, J. M. and Fernández-Martínez, M. Near-Earth object hazardous impact: A Multi-Criteria Decision Making approach. *Sci. Rep.* **6**, 37055; doi: 10.1038/srep37055 (2016).

**Publisher's note**: Springer Nature remains neutral with regard to jurisdictional claims in published maps and institutional affiliations.

## Supplementary Material

Supplementary Information

## Figures and Tables

**Table 1:** Scale of valuation in the pairwise comparison process.

*Labels*	*Verbal judgments of preferences between i and j criteria*	*Saaty’s scale*
(*EI*)	*C*_*i*_ and *C*_*j*_ are equally important	1
(*S* + *I*)	*C*_*i*_ is slightly more important than *C*_*j*_	3-1/3
(*St* + *I*)	*C*_*i*_ is strongly more important than *C*_*j*_	5-1/5
(*VSt* + *I*)	*C*_*i*_ is very strongly more important than *C*_*j*_	7-1/7
(*Ex* + *I*)	*C*_*i*_ is extremely more important than *C*_*j*_	9-1/9

This scale is used in AHP methodology to carry out comparisons between pairs of criteria lying in the same level of a hierarchy.

**Table 2:** Criteria weights to assess hazardous NEOs through an experts’ homogeneous aggregation via arithmetic average.

*Criteria*	*Importance weight (%*)	*Rank*
***C***_***1***_	21.2	2
***C***_***2***_	20.2	3
***C***_***3***_	6.6	5
***C***_***4***_	5.3	7
***C***_***5***_	5.8	6
***C***_***6***_	23.1	1
***C***_***7***_	17.8	4

**Table 3:** Top 10 most hazardous NEOs according to TOPSIS ranking.

*Alternatives*	*R*_*i*_ (*TOPSIS*)	*Ranking*	*Year 1*^*st*^ *hypothetic impact*
**410777 (2009 FD)**	0.4857	**1**	**2185**
**2011 SR52**	0.4410	**2**	**2034**
2015 HV182	0.2963	3	2016
2010 MA113	0.2786	4	2033
2014 NZ64	0.2432	5	2017
2008 VS4	0.1996	6	2016
101955 Bennu (1999 RQ36)	0.1870	7	2175
2014 MO68	0.1781	8	2017
2007 KO4	0.1731	9	2022
29075 (1950 DA)	0.1510	10	2880

**Table 4:** Decision matrix involving the 10 Top-ranked NEOs.

Alternatives	*Criteria*						
	*C*_*1*_	*C*_*2*_	*C*_*3*_	*C*_*4*_	*C*_*5*_	*C*_*6*_	*C*_*7*_
410777 (2009 FD)	7	1.60E-03	15.87	22.10	0.160	−1.78	1.40E + 02
2011 SR52	4	7.60E-10	13.55	15.60	0.054	−4.35	8.60E + 05
2015 HV182	511	7.10E-07	7.82	21.70	0.154	−4.07	1.20E + 02
2010 MA113	469	5.20E-06	3.06	23.40	0.078	−4.47	2.00E + 01
2014 NZ64	388	2.10E-06	6.61	22.50	0.108	−4.25	3.20E + 01
2008 VS4	304	6.20E-07	8.15	24.10	0.050	−5.25	5.00E + 00
101955 Bennu (1999 RQ36)	78	3.70E-04	5.99	20.20	0.490	−1.71	1.20E + 03
2014 MO68	262	1.50E-06	8.36	23.50	0.067	−5.06	1.00E + 01
2007 KO4	248	4.20E-06	13.71	23.30	0.075	−4.43	2.10E + 01
29075 (1950 DA)	1	1.20E-04	14.10	17.60	1.300	−1.42	7.50E + 04

**Table 5:** Top-rated alternatives by TOPSIS provided that all the criteria are chosen to be equally relevant.

*Alternatives*	*Weights through experts group* (Table 2)		*All the criteria with the same weight*	
	*R*_*i*_ (*TOPSIS*)	*Ranking*	*R*_*i*_ (*TOPSIS*)	*Ranking*
410777 (2009 FD)	0.4857	**1**	0.4479	2
2011 SR52	0.4410	**2**	0.4526	1
2015 HV182	0.2963	3	0.2646	4
2010 MA113	0.2786	4	0.2459	5
2014 NZ64	0.2432	5	0.2144	6
2008 VS4	0.1996	6	0.1748	10
101955 Bennu (1999 RQ36)	0.1870	7	0.1903	7
2014 MO68	0.1781	8	0.1558	12
2007 KO4	0.1731	9	0.1538	13
29075 (1950 DA)	0.1510	10	0.2732	3

**Table 6:** Comparing the top-rated alternatives including the PR (the two columns on the left) vs. excluding it.

*Alternatives*	*Assigning PR 25% of the overall relevance and remaining criteria being equally weighted*		*Weights provided by experts (c.f.* Table 2)	
	*R*_*i*_ (*TOPSIS*)	*Ranking*	*R*_*i*_ (*TOPSIS*)	*Ranking*
2013 NH6	0.5882	1	0.0452	71
410777 (2009 FD)	0.2881	2	0.4857	1
2011 SR52	0.2829	3	0.4410	2
99942 Apophis (2004 MN4)	0.2225	4	0.0867	19
29075 (1950 DA)	0.1860	5	0.1510	10
2015 HV182	0.1660	6	0.2963	3
2010 MA113	0.1530	7	0.2786	4
101955 Bennu (1999 RQ36)	0.1398	8	0.1870	7
2014 NZ64	0.1318	9	0.2432	5
2005 GV190	0.1082	10	0.0705	31

The calculations have been carried out via the TOPSIS approach in both cases.

**Table 7:** Random Index (*RI*) for matrix orders from 1 to 15 where *n* represents the number of compared criteria^31^.

*n*	*1–2*	*3*	*4*	*5*	*6*	*7*	*8*	*9*	*10*
***RI***	0.00	0.5247	0.8816	1.1086	1.2479	1.3417	1.4057	1.4499	1.4854
***n***	***11***	***12***	***13***	***14***	***15***				
***RI***	1.5140	1.5365	1.5551	1.5713	1.5838				

**Table 8:** Decision matrix for a TOPSIS approach.

	*w*_*1*_	*w*_*2*_	*…*	*w*_*j*_	*…*	*w*_*n*_
	*C*_*1*_	*C*_*2*_	*…*	*C*_*j*_	*…*	*C*_*n*_
*A*_*1*_	x_11_	x_12_	…	x_1j_	…	x_1n_
*A*_*2*_	x_21_	x_22_	…	x_2j_	…	x_2n_
*…*	…	…	…	…	…	…
*A*_*m*_	x_m1_	x_m2_	…	x_mj_	…	x_mn_

*x*_*ij*_ refers to the performance score of alternative *A*_*i*_ with respect to criteria *C*_*j*_ and *W* = [*w*_*1*_,*w*_*2*_,…,*w*_*n*_] denotes the weight vector associated with these criteria.
