# Supplementary material for: Near-Earth object hazardous impact: A Multi-Criteria Decision Making approach
Source: Sci Rep. 2016 Nov 16;6:37055. doi: 10.1038/srep37055 (PMC5111055; doi:10.1038/srep37055)
Supplement: Supplementary Information [file srep37055-s1.pdf]

Near-Earth object hazardous impact: A Multi-Criteria Decision Making approach

J. M. Sánchez-Lozano[(1)], M. Fernández-Martínez[(1)]

[(1)] University Centre of Defence at the Spanish Air Force Academy, MDE-UPCT.

C/Coronel López Peña, s/n. 30720 Santiago de la Ribera, Murcia (SPAIN)

Appendix A. TOPSIS ranking to assess the most hazardous NEOs. The objects appeared in the list below have been rated by descending order of their TOPSIS score.

Alternatives	R_i *(TOPSIS)*	*Alternatives*	R_i *(TOPSIS)*	*Alternatives*	R_i *(TOPSIS)*
410777 (2009 FD)	0.4857	2015 BS516	0.0679	2011 BA60	0.0462
2011 SR52	0.4410	2008 ST7	0.0679	2015 FA345	0.0457
2015 HV182	0.2963	2008 PK9	0.0675	2013 NH6	0.0452
2010 MA113	0.2786	2013 WM	0.0674	2014 QF33	0.0444
2014 NZ64	0.2432	2006 CD	0.0669	2008 DA4	0.0444
2008 VS4	0.1996	2010 CA55	0.0669	2001 SB170	0.0438
101955 Bennu (1999 RQ36)	0.1870	2007 WP3	0.0649	2007 VH189	0.0433
2014 MO68	0.1781	2012 ES10	0.0649	2012 PK24	0.0425
2007 KO4	0.1731	2006 JE	0.0641	2009 WQ25	0.0411
29075 (1950 DA)	0.1510	2013 JA17	0.0640	2008 XC1	0.0406
1994 WR12	0.1254	2004 ME6	0.0639	2012 SG58	0.0404
2007 FT3	0.1251	2002 MN	0.0633	2005 GQ33	0.0383
2015 YV20	0.1041	2011 VG9	0.0627	2008 KN11	0.0381
2005 TM173	0.1005	2002 GM5	0.0625	2012 CR	0.0354
1979 XB	0.1000	1996 TC1	0.0616	2006 WK130	0.0350
2015 ME131	0.0996	2015 DA54	0.0612	2005 CC37	0.0345
2001 CA21	0.0970	2011 YV62	0.0610	2014 JU79	0.0316
2010 JA43	0.0892	2012 BG96	0.0602	2010 LF64	0.0311
99942 Apophis (2004 MN4)	0.0867	2007 TC14	0.0602	2009 XQ2	0.0304
2008 UB7	0.0864	2010 LJ68	0.0596	2001 UO	0.0287
2008 UV99	0.0839	2009 BR5	0.0591	2014 HN197	0.0251
2005 NX55	0.0799	2000 WJ107	0.0584	2012 UU68	0.0251
2011 BT59	0.0798	2002 RB182	0.0568	2007 WT3	0.0241
2005 ED224	0.0770	2010 KJ37	0.0566	2009 WZ53	0.0234
2010 DJ77	0.0763	2014 NJ65	0.0560	2012 BN123	0.0231
2008 FF5	0.0750	2013 VJ13	0.0553	2014 OO6	0.0218
2010 CR5	0.0726	2004 GE2	0.0552	2010 LV108	0.0198
2012 QD8	0.0724	2006 QN111	0.0547	2014 HM199	0.0193
2010 WC9	0.0709	2011 KF36	0.0547	2014 MG68	0.0179
2014 MV67	0.0706	1999 RZ31	0.0529	2014 MR67	0.0170
2005 GV190	0.0705	2016 AY193	0.0518	2014 OQ392	0.0126
2010 XB73	0.0699	2006 CM10	0.0517	2007 WW3	0.0112
2008 EX5	0.0697	2005 WG57	0.0483	2013 UO5	0.0097
2011 XC2	0.0689	2001 UD5	0.0465		
